# A Speech

**Published:** 1869-06

**Authors:** 


### "A SPEECH."

At the close of one of M. Blondin's rope exhibitions at Niagara, as he came to shore, pale and fatigued, hundreds saluted him with the cry of "*a speech!* A SPEECH!!" To them it mattered not that he had performed on the rope, many, and more daring feats than he had promised. Unless he could make a speech, then and there, he was in their eyes an impostor. And they were not singular in their sentiments. So universally has this idea of speech making taken hold of us, that we no longer hail each other with the good old interjection, Ho! But instead, we call out, SAY! Indeed, it is rumored that at a late "*seance*," the ghost of a grand old prophet was called for, and instead of his sublime, Ho! every one that thirsteth," he rapped out, "*Say!* Mister, 'll y've somethin' to drink?" And so imperative has this sentiment become that most persons, when called on, by voice or circumstance, feel obliged to "say" whatever they are able to say at the time, whether it be sense or nonsense. And if our worthy President and Lieutenant General succeed in persuading the American people that making speeches is not essential to good manners, reputation, or success, they will be entitled to the lasting gratitude of the nation. We have been led to these reflections by observing that a "friend of our better days" has had a speech forced out of him when there was none in him, or at least none on the subject under consideration.

Elsewhere we have noticed that a Dental College has been organized at Chicago, a step in the right direction, if the proper *stand* is taken after the step. In the "Missouri Dental Journal" we find an address, on the occasion, by one of the newly appointed trustees, R. L. Rea, M. D. We knew Dr. R. when he was a ploughboy, and he was a good ploughboy; knew him when a school-teacher, and he was good there; when he was a physician, a professor, etc. And we have watched him with interest through all; and we happen to know that he is totally unacquainted with the formation of and course of instruction usual in Dental Colleges, and if we had not known it, this abortive address, a result of Mrs. Custom's periodical pills, perhaps, would have given us the information.

As all men do when forced to speak on a definite subject without a knowledge of the facts relating to it, our friend starts out hit or miss; and in reading his address we are reminded of the precaution of the Irish boy at the shooting-match. So wild were the aims, he concluded the only place of safety was in front of the target.

The doctor gives reasons for the new enterprise. The first is Chicago; and we give in to that, for there she stands. Another is, "the palpable necessity there is for a higher standard of Dental education." Certainly. But shouldn't Chicago start a few more medical colleges? For the standard of medical education in America is far below that of Dental education. We have had vastly better opportunities for making a correct comparison than Dr. R.; and we are sorry that truth requires such an assertion.

The address states farther, "It is notoriously true, that a large majority of the members of the Dental profession are not only ignorant of the primary and essential facts in medicine, but are allowed to graduate from Dental Colleges in deplorable ignorance of the principles of medical science." And if the word *medical* were substituted for "Dental" in the above sentence, it would be quite as elegant and certainly as truthful; and we can join our friend, let it be stated either way, in saying, "This is not as it should be."

And this speaker who has had no opportunity whatever to learn anything in regard to the course of instruction in any Dental school, and who knows quite as little in regard to the tastes and habits of Dental students, goes on to say, "The apt and elegant apparatus needed, the perfect and beautiful adjustment of parts occupies so large a portion of the time of the student, that they soon engross his entire attention, and supplant the proper groundwork of anatomy, physiology, pathology, etc." Now, if he had inquired of a fellow *alumnus* who was a physician when he was a schoolboy, and who has been a Dental teacher longer than he has been a physician, he would have learned that Dental students are inclined to *neglect* the mechanical, for the sake of the "groundwork" sciences to which he refers. And he would have learned that Dental mechanism, though admitted to be of importance, receives but a small portion of time and attention in any Dental school.

"Comparisons are odious;" but in what medical school is each candidate subjected to from two to five hours written examination on each department, after a rigid oral examination, and a winter's "quiz," with the condition that seventy-five per cent. of correct answers are required on each department? And how many medical schools make a satisfactory examination in the junior studies a requisite to admission to the senior course? And how much did medical schools do for Dental Surgery in the years preceding the era of Dental schools? Not half the medical graduates that we have had occasion to talk with on the subject, know a permanent from a temporary molar. Scores of them have brought their children to us to have the six-year molars extracted, under the impression that they were temporary, and would soon be replaced.

The first Dental school, in the first ten years of its existence, did more to advance and improve Dental surgery than all the medical schools in the worl l have done since the dawn of science. Yet this is one of the institutions so ungrammatically denounced in the address.

Another Dental school has had among its teachers several of the most renowned and efficient professors of the speaker's *alma mater*, and has now a goodly proportion of her older *alumni* in its faculty, and they all *knew*, from the start, what this new-fledged trustee begins already to suspect, viz.: "that it should be the duty of Dental colleges to require something more than a simple knowledge of the jaws," etc.

The Dental schools, as a general thing, are conducted by men well versed in medical science. Many of them are graduates from medical schools, but these are not superior to those who are not. And physicians should remember that society is indebted to Dentists, as such, for anæsthesia, both general and local, while, so far as Dental surgery and therapeutics are concerned, it owes the medical profession for the turnkey and mercurial ptyalism.

Now, if Chicago will carry on a good Dental school, we shall rejoice. There are some in the board of trustees that could have said as much in favor of a Chicago school without murdering both grammar and rhetoric in denouncing others; and it was cruel in them to let our friend expose himself as he did.

A sensitive student had been reprimanded and appeared vexed. A comrade tried to console him. He replied, "O, I don't care for the reprimand; but I hate to be scolded in *bad grammar*, by a PROFESSOR.

Our friend never talks that way on a subject he understands.

